# Large-Scale Microcarrier Culture of Chinese Perch Brain Cell for Viral Vaccine Production in a Stirred Bioreactor

**DOI:** 10.3390/vaccines9091003

**Published:** 2021-09-08

**Authors:** Xia Luo, Yinjie Niu, Xiaozhe Fu, Qiang Lin, Hongru Liang, Lihui Liu, Ningqiu Li

**Affiliations:** Guangdong Province Key Laboratory of Aquatic Animal Immune Technology, Key Laboratory of Fishery Drug Development, Pearl River Fisheries Research Institute, Chinese Academy of Fishery Sciences, Ministry of Agriculture and Rural Affairs, Guangzhou 510380, China; luoxia@prfri.ac.cn (X.L.); nyj@prfri.ac.cn (Y.N.); fuxiaozhe@prfri.ac.cn (X.F.); linq@prfri.ac.cn (Q.L.); hrliang@prfri.ac.cn (H.L.); liulh@prfri.ac.cn (L.L.)

**Keywords:** CPB cell line, microcarrier, Cytodex 1, suspension culture, bioreactor

## Abstract

Mandarin fish (*Siniperca chuatsi*) is one of the important cultured fish species in China. Infectious spleen and kidney necrosis virus (ISKNV) and *Siniperca Chuatsi* rhabdovirus (SCRV) have hindered the development of mandarin fish farming industry. Vaccination is the most effective method for control of viral diseases, however viral vaccine production requires the large-scale culture of cells. Herein, a suspension culture system of Chinese perch brain cell (CPB) was developed on Cytodex 1 microcarrier in a stirred bioreactor. Firstly, CPB cells were cultured using Cytodex 1 microcarrier in 125 mL stirring flasks. With the optimum operational parameters, CPB cells grew well, distributed uniformly, and could fully cover the microcarriers. Then, CPB cells were digested with trypsin and expanded step-by-step with different expansion ratios from the 125 mL stirring bottle to a 500 mL stirring bottle, and finally to a 3-L bioreactor. Results showed that with an expansion ratio of 1:3, we achieved a high cell density level (2.25 × 10^6^ cells/mL) with an efficient use of the microcarriers, which also confirmed the data obtained from the 125 mL stirring flask. Moreover, obvious cytopathic effects (CPE) were observed in the suspended CPB cells post-infection with ISKNV and SCRV. This study provided a large-scale culture system of CPB cells for virus vaccine production.

## 1. Introduction

Mandarin fish (*Siniperca chuatsi*) is one of the important cultured fish species in China. In recent years epidemic diseases, mainly caused by infectious spleen and kidney necrosis virus (ISKNV) and *Siniperca chuatsi* rhabdovirus (SCRV), have seriously hindered the development of the mandarin fish farming industry [1,2,3]. Vaccination is the most effective method for control of viral diseases. In China, Dong [4] and Fu [5] have developed the sensitive cell lines mandarin fish fry (MFF-1) and Chinese perch brain (CPB) for ISKNV and SCRV multiplication. Subsequently, several effective inactivated or live vaccines against the mentioned viruses have been developed. A research group from Sun Yat-sen University developed an inactivated ISKNV vaccine and have obtained the corresponding veterinary drug certificate (No. 75) in 2020. Zhang et al. [6] developed an inactivated ISKNV vaccine with chitosan as the adjuvant, which can play a significantly protective role against ISKNV infections. Zhang et al. [7] reported a clonal *Micropterus salmoides* rhabdovirus Sanshui-7 (MSRV-SS-7) strain as a potential live vaccine candidate for Chinese perch against SCRV disease. Recently, our laboratory developed an ISKNV&SCRV bivalent inactivated vaccine which can provide simultaneous protection against ISKNV and SCRV infections with a protection rate of more than 90% and 80%, respectively [8].

In the current race for the virus vaccine production, microcarrier and suspension culture systems are amongst the most promising techniques available. Since the introduction of microcarriers by van Wezel in 1967, their use in the production of many biologicals by anchorage-dependent mammalian cells has largely supplanted traditional roller bottle facilities [9]. As the microcarrier bead has a large surface area for cell attachment, therefore, it can produce a larger number of cells compared with the conventional monolayer culture [10,11]. Moreover, the benefits of using microcarrier cultures for vaccine production are numerous, such as labor-, consumables- and space-savings [12]. Yang et al. [13] described large-scale cultivation processes for HEK293T cells and Vero cells in XDR-50 and XSR-200 bioreactors. Sousa et al. [14] developed a scalable bioprocess for Peste des Petites Ruminants virus (PPRV) vaccine production in Vero cells using SFM, microcarrier technology in STB, in-situ cell detachment from microcarriers and perfusion. Recently, Vero cells have been successfully adapted to grow in suspension in serum-free media [15,16]. Fujii et al. [17] fabricated an ultrasonic irradiation system and used CHO cells as a model example, and they found the culture time was reduced by 14% when the cell number increased 1000-fold.

**Figure 1:**
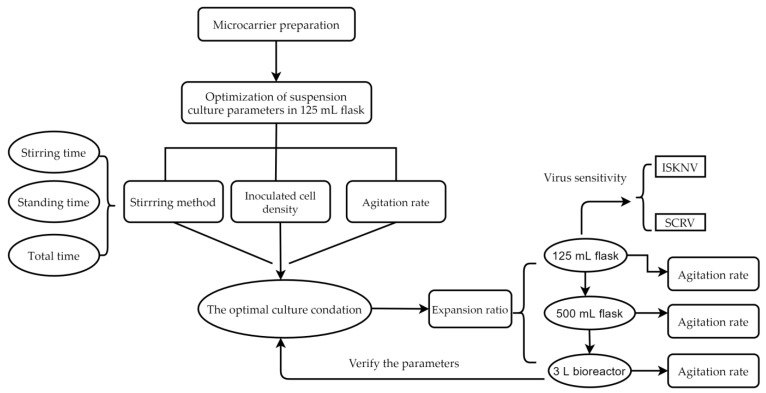
Flow diagram for the experimental process.

However, roller bottle culture and cell factories are still the main techniques used for large-scale production of vaccines for mandarin fish at present. Despite the advantages described above, no attempt has been reported in the literature describing the use of cells from mandarin fish so far. CPB cells which are sensitive to both ISKNV and SCRV have been developed by Fu et al. [5] in our laboratory. To overcome the deficiencies of the roller bottle culture method [18], this study aimed to develop an easy scalable process for the production of CPB cells for ISKNV and SCRV proliferation with high productivity. In this paper, the suspension culture of CPB cells was developed using microcarrier Cytodex 1. Some cultivation parameters were optimized, including the agitation method at the cell attachment stage, density of inoculated cells, microcarrier concentration, expansion ratio and agitation rate in different culture systems (Figure 1). This study provided technical parameters for scale production of CPB cells for ISKNV and SCRV vaccine production.

## 2. Materials and Methods

### 2.1. Microcarrier Preparation

Cytodex 1 from GE HealthCare (Uppsala, Sweden) was used in this study. Microcarriers were prepared and sterilized according to the manufacturer’s instructions. Briefly, all microcarriers were hydrated in Ca^2^^+^ and Mg^2^^+^ free phosphate-buffered saline (PBS), washed twice with fresh PBS, autoclaved, and equilibrated in Leibovitz’s L15 culture medium with 10% foetal bovine serum (FBS) for at least 24 h prior to use in microcarrier-assisted culture experiments [19].

### 2.2. Optimization of Suspension Culture Parameters

#### 2.2.1. Determination of the Appropriate Stirring Method

CPB cells were inoculated with an initial concentration of 30 living cells/microcarrier, and the Cytodex 1 density was 3 g/L. Cultures were carried out in 125 mL spinner flasks containing 50 mL of culture medium. Different intermittent stirring methods with a low agitation rate during the early stages of cell attachment were tested (Table 1). Once the intermittent agitation finished, samples were collected immediately to determine cell number with crystal violet staining method as described by Trabelse [20]. The optimal stirring method was determined by the attachment ratio and empty-loading ratio (when the cells on the microcarrier were below 5):

Attachment ratio = Number of cells when the intermittent stirring finished/Number of inoculated living cells

#### 2.2.2. Determination of the Optimal Inoculated Cell Density

With 3 g/L Cytodex 1 and half volume of the culture medium, experiments with different inoculation densities of cells ranging from 20 to 50 living cells/microcarrier were carried out. Firstly, CPB cells were stirred intermittently at an agitation rate of 40 r/min for 3 min every 42 min for 5 h (3 min/42 min/5 h) during the attachment stage, and then the culture medium was supplemented to work volume. Samples were collected daily at a homogeneously suspended state. Growth status of cells on microcarrier was observed under the microscope, and number of the cells was counted after stained with crystal violet. All assays were performed in triplicate and average cell number was used for the cell growth curve with different inoculation density of living cells.

#### 2.2.3. Determination of the Optimal Microcarrier Concentration

To determine effects of concentration of Cytodex 1 on CPB proliferation, four different concentrations included 2, 3, 4 and 5 g/L were tested. The same density of living cell inoculation and stirring method at cell attachment stage were used as described above. Cell growth kinetics with different microcarrier concentrations was compared with the previous methods.

#### 2.2.4. Determination of the Appropriate Cell Amplification Ratio in Different Culture Systems

**Figure 2:**
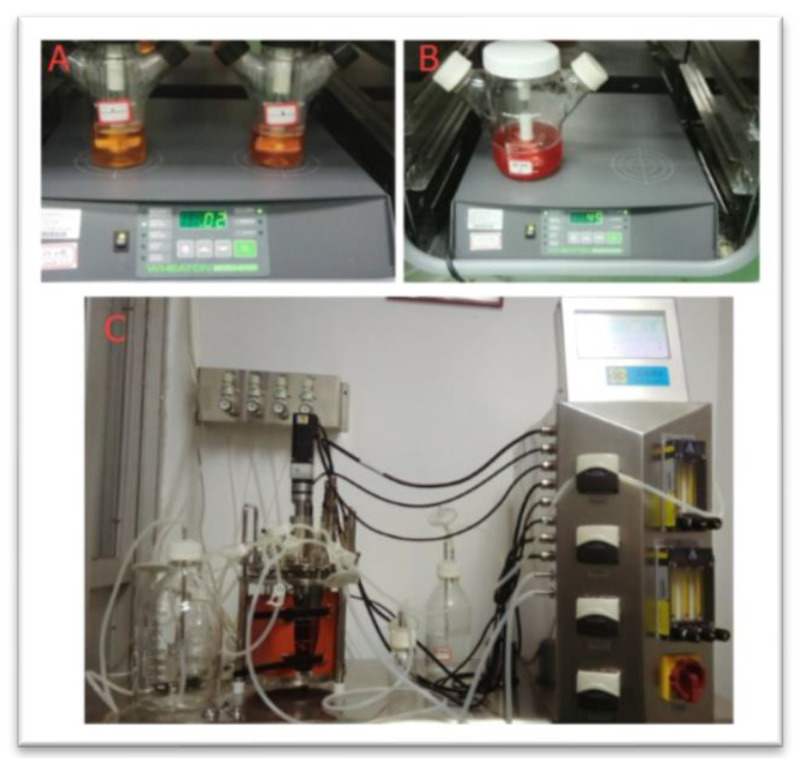
Different suspension culture systems. (**A**) The 125 mL stirring flask; (**B**) the 500 mL stirring flask; (**C**) the 3 L bioreactor.

When grown to the stable phase, CPB cells were digested with 0.25% trypsin and amplified step-by-step with different expansion ratios from the 125 mL stirring bottle to 500 mL stirring bottle, and at last to 3 L bioreactor (Figure 2). Cells were inoculated at the concentration of 30 cells/microcarrier with 3 g/L Cytodex 1 microcarriers, and then samples were collected and counted after being stained with crystal violet when cells reached the maximum number. Then cells were expanded as follows: stirring is stopped to let the microcarriers settle down, the supernatant is removed, the cells are washed twice with PBS, and then preheated trypsin is added according to standard protocols using an agitation rate of 50 r/min. Samples were collected and observed under the microscope. When cells more than 70% detached from the microcarriers, the digestion procedure was immediately stopped by adding L15 containing 10% FBS. With the same microcarrier concentration, the expansion ratios tested included 1:2, 1:3, 1:4 and 1:5 between old and new microcarriers. Five hours later, samples were collected and counted after being stained with crystal violet to determine the cell viability. All assays were performed in triplicate. Cell number was counted daily and the mean values were used for the cell growth curve. Survival rates were estimated using the following equation:

Survival rate= (Cell number before amplification /Cell number at the fifth hour after amplification) ×100%

#### 2.2.5. Determination of the Optimal Agitation Rate in Different Culture Systems

An expansion ratio of 1:3 was used from 125 to 500 mL flasks and then to a 3 L bioreactor. First at the cell attachment stage, the culture system was stirred intermittently (3 min/42 min/5 h) at 28 °C with a magnetic stirring apparatus. Then, three different agitation rates (30, 40 and 50 r/min for 125 mL flask, 30, 45 and 60 r/min for 500 mL flask, 40, 55 and 70 r/min for the 3 L bioreactor) were set, respectively, according to the volume of the culture system. Samples were collected and observed under the microscope every day. The number of the cells was counted after being stained with crystal violet, and mean values were used for cell growth curves with different agitation rate.

### 2.3. Virus Sensitivity

To test the virus sensitivity of the suspended CPB cells, ISKNV and SCRV were inoculated into the cells, respectively, after overnight culturing on the macrocarriers in a 125 mL flask. Before inoculation, recirculation and agitation were stopped and microcarriers were allowed to settle. Then the supernatant was removed, the cells were washed twice with the same volume of L15. ISKNV and SCRV were inoculated at the multiplicity of infection (MOI) of 1 and 0.01 in half of the working volume filled with incubated buffer without FBS, respectively. Intermittent stirring was started at 40 r/min for 3 min every 12 min for 2 h during the early infection stage, then the bioreactor volume was supplemented to the final volume of 100 mL with 5% FBS. To observe the CPE, samples were collected and observed daily under the microscope.

### 2.4. Statistical Analysis

All of the experiments in this paper were performed in triplicate and each experiment was repeated three times. The data were expressed as means ± standard deviation (*n* = 3). All the statistical analyses were done with the SPSS ver. 20 statistical software package (IBM Corp, Armonk, NY, USA).

## 3. Results

### 3.1. Stirring Method at the Attachment Stage of CPB Cells

**Figure 3:**
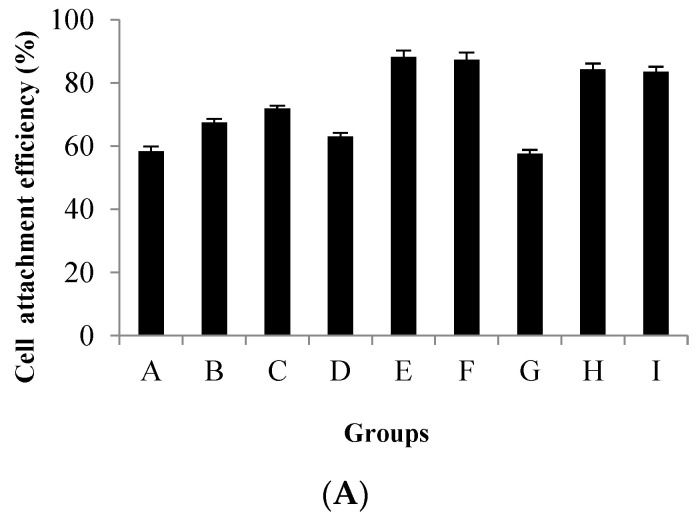

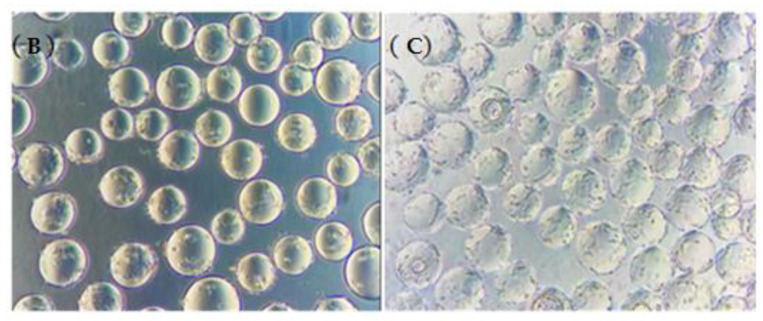
Cell attachment 5 h post inoculation. (**A**) Cell attachment efficiency with different agitation methods (from group A to H). Bars show the mean ± SD, *n* = 3; (**B**,**C**) cell attachment status of group H (**B**) and group E (**C**) observed under the phase contrast microscope (10×).

The cell attachment ratio on Cytodex 1 was studied with different stirring methods from group A to I. Figure 3 indicates that the attachment ratio was only around 60% in group A, D and G, obviously lower than groups B, C, E, F, H and I. According to the results from group A to I, we found that increase in intermittent stirring time improved the attachment ratio. However, there was not a distinct difference between 5 h and 8 h. Although cell attachment efficiency (i.e., 80%) was high in groups H and I (3 min/57 min), the empty-loading ratio was more than 10%, and meanwhile the cells were distributed heterogeneously. When the stirring method in group E (3 min/42 min/5 h) was carried out, CPB cells were distributed homogeneously on Cytodex 1 (Figure 3B), and also with a high attachment efficiency (i.e., 88.2%) and low empty-loading ratio (i.e., less than 10%).

### 3.2. Determination of the Optimal Inoculated Cell Density

**Figure 4:**
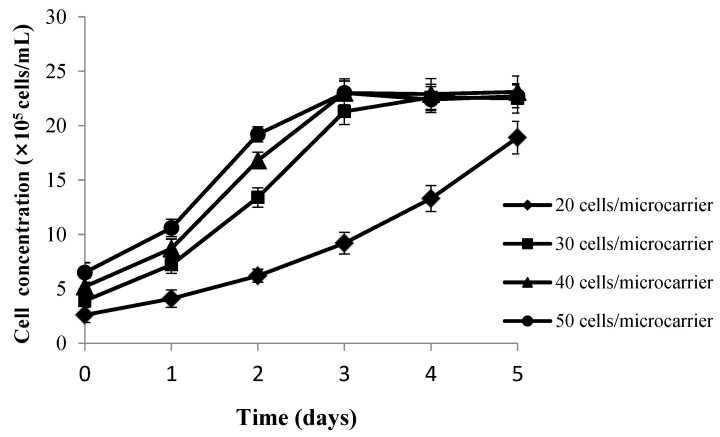
Growth curve of CPB cells at different inoculation densities. Bars show the mean ± SD, *n* = 3.

As shown in Figure 4, cells grew slowly and didn’t achieve a plateau with an inoculation density of 20 cells/microcarrier. When the inoculation density was increased to 40 living cells/microcarrier, cells were distributed heterogeneously with concomitant agglomeration. When the inoculation density was further increased to 50 living cells/microcarrier, further agglomeration occurred, and the cell density reached the highest level at the third day and then cells started to detach from the microcarrier. While the inoculation density was 30 living cells/microcarrier, cells were distributed relatively homogeneously, concomitantly with rare agglomeration. Furthermore, the empty-loading ratio was less than 5%, and the highest cell density reached was about 2.3 × 10^6^ cell/mL. Thus, 30 living cells/microcarrier was selected as the optimal inoculation cell density.

### 3.3. Microcarrier Concentration

**Figure 5:**
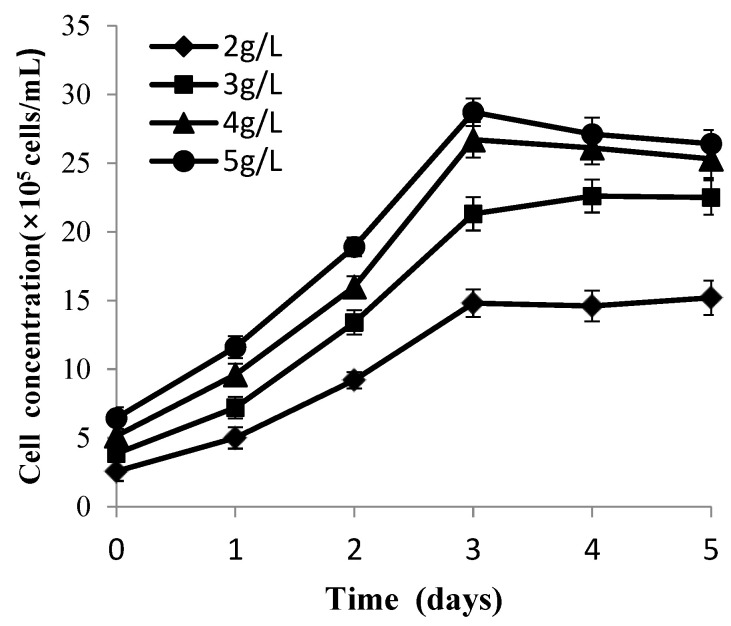
Growth curve of CPB cells at different microcarrier concentrations. Bars show the mean ± SD, *n* = 3.

The data depicted in Figure 5 showed that CPB cells of the four groups all grew well and achieved a plateau at the third day. With different densities of Cytodex 1 ranging from 2 to 5 g/L, the maximal cell concentration reached 1.48 × 10^6^, 2.26 × 10^6^, 2.67 × 10^6^ and 2.87 × 10^6^ cell/mL, respectively, so the higher the density of Cytodex 1, the higher the concentration of the cells obtained was. In order to optimize the density of Cytodex 1, cell expansion fold and daily cell specific growth rate were determined. The data (Table 2) indicated that lower daily cell specific growth rate appeared in the higher density of Cytodex 1 group and the lower cell expansion fold group. Cells in the 4 and 5 g/L microcarrier concentration groups, both achieved a highest concentration level at the third day and then declined. In addition, detached cells and fragments were also observed in the suspension liquid. Therefore, the optimal microcarrier concentration for CPB cell attatchment is 3 g/L.

### 3.4. Determination of the Appropriate Cell Expansion Ratio between Different Culture Systems

**Figure 6:**
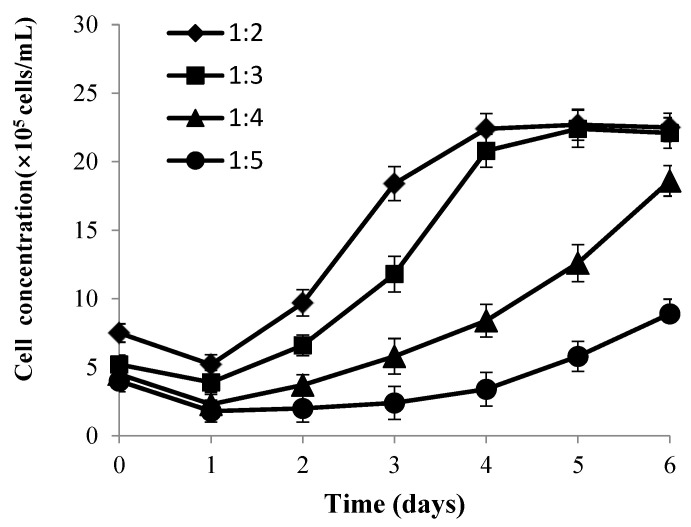
Growth curve of CPB cells at different expansion ratios. Bars show the mean ± SD, *n* = 3.

When CPB cells grow to a monolayer on Cytodex 1, expansion is necessary. The concentration of the cells at the plateau showed little difference (less than 10%) among different batches under the same culture conditions, proving fine repeatability. Data depicted in Figure 6 shows that the cell densities with all of the expansion ratios declined on the first day. 

When the expansion ratio was 1:2 and 1:3, cells reached the stable phase at the fourth day with a maximal concentration of 2.27 × 10^6^ and 2.24 × 10^6^ cells/mL, respectively. When the ratio was 1:4, cells proliferated relatively slowly, and it couldn’t grow to a monolayer even until the sixth day. When the ratio was increased to 1:5, the specific growth rate was low with a long incubation period, and cells still proliferated slowly even on the sixth day. The number of harvested cells at the ratio of 1:3 was larger than at the ratio of 1:2. In conclusion, the expansion ratio at 1:3 was the cost-optimal choice.

### 3.5. Determination of Appropriate Agitation Rate in Different Culture Systems

**Figure 7:**
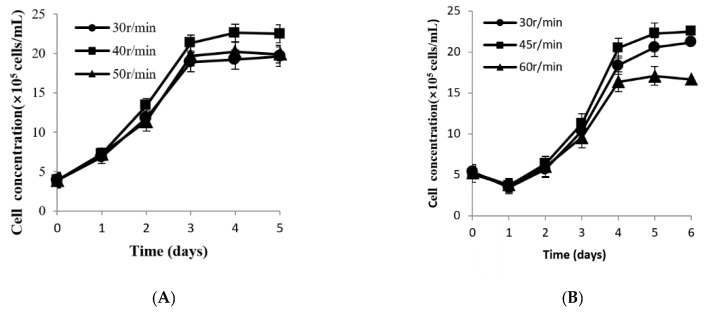

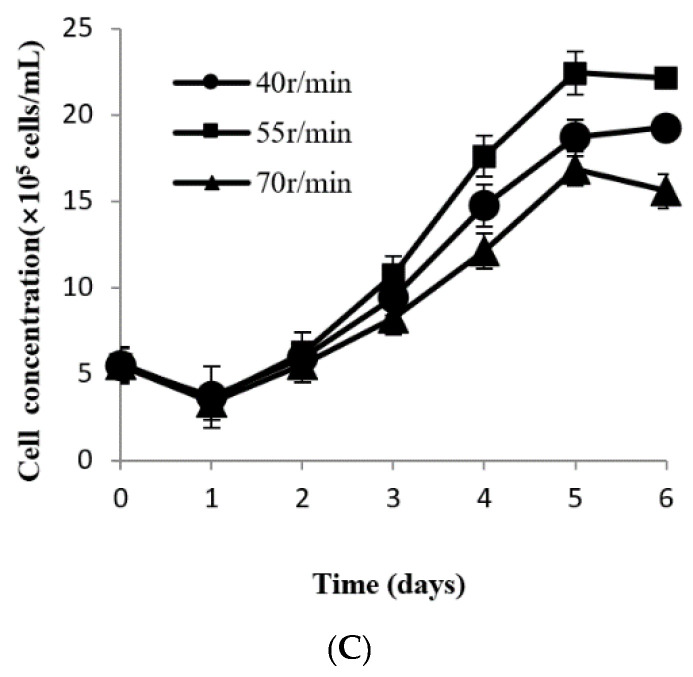
Growth curve of CPB cells at different agitation rates. (**A**) The 125 mL flask; (**B**) the 500 mL flask; (**C**) the 3 L bioreactor. Bars show the mean ± SD, *n* =3.

The cell expansion rate gradually increased in 125 mL flasks with 30–40 r/min as the agitation rate (Figure 7A). However, when the agitation rate exceeded 40 r/min, the maximum concentration of the cells started to decline. The empty-loading ratio was high with the agitation rate of 30 r/min. Cells were distributed homogeneously when the agitation rate was 40 r/min. When increased to 50 r/min, a small amount of cells detached from the microcarriers, and with a heterogeneous distribution. Moreover, the cell concentration with an agitation rate of 40 r/min was higher compared to the 30 r/min and 50 r/min groups. Therefore, 40 r/min is the optimal agitation rate for CPB culture in 125 mL flask. With a similar method, optimal agitation rates of 45 r/min and 55 r/min were determined for the 500 mL flask (Figure 7B) and 3 L bioreactor, respectively (Figure 7C).

### 3.6. Comparison of Suspension Culture and Traditional Culture Process

**Figure 8:**
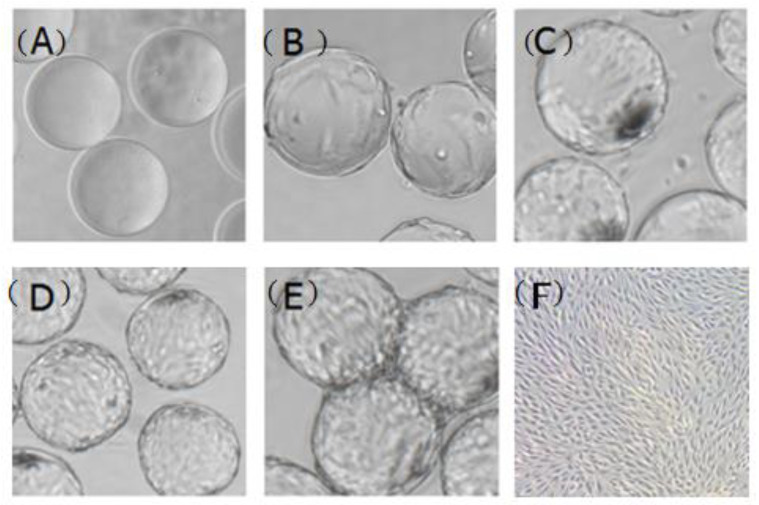

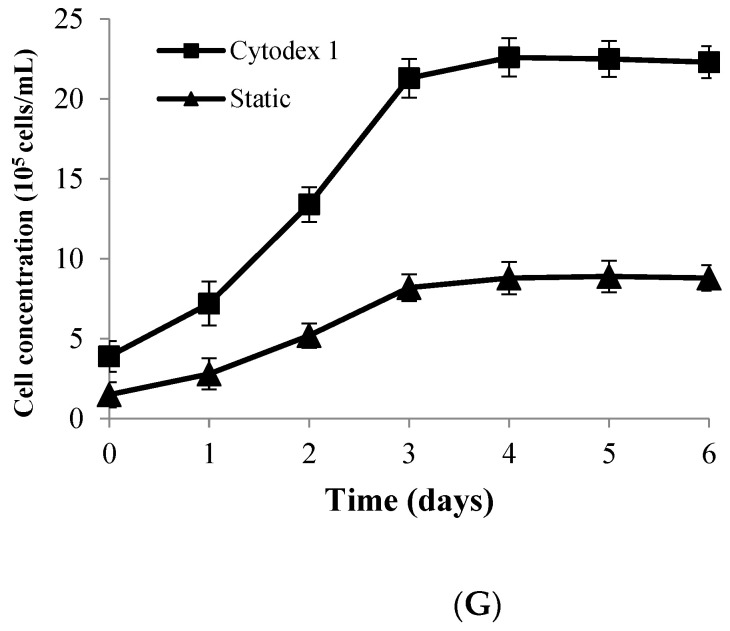
Characteristics of CPB cells expanded under static and suspended culture conditions. (**A**–F) Morphology of the cells grown on Cytodex1 and culture plate (20×) (**A**) Bare microcarrier; (**B**) Cell attachment on the first day; (**C**) 50% of the microcarriers were coated with CPB cells on the second day; (**D**) Cells grew to a single-layer on the third day; (**E**) “cell bridge” appeared when cells were fully attached on Cytodex1. (**F**) CPB cells in the static culture plate; (**G**) Growth curve of CPB cells expanded under static and suspended culture conditions. Bars show the mean ± SD, *n* =3.

Using the optimal parameters, CPB cells grew well on Cytodex 1. After inoculation, cells attached steadily on the microcarriers 5 h later (Figure 8B). On the second day, about 50% of the microcarriers were coated with CPB cells (Figure 8C). On the third day, cells grew to a single-layer (Figure 8D) and then cell bridges appeared when the cells were attached fully on Cytodex 1 (Figure 8E). When CPB cells were cultured in a static system, cells were inoculated at a density of 1.5 × 10^5^ cells/mL, and achieved a maximum density of 8.9 × 10^5^ cells/mL on the fourth day, which was equal to 1.85 × 10^5^ cells/cm^2^ (Figure 8F, G). While CPB cells were cultured with the suspended system, the cell density achieved a highest level of 2.26 × 10^6^ cells/mL (Figure 8G), about 2.5-fold higher than with the static system. There are 4.3 × 10^6^ microcarriers for each gram of Cytodex 1 providing a superficial area of around 4400 cm^2^. Theoretically, the number of living cells should be 189.3 on every Cytodex 1 when grown as a monolayer, which is in line with the actual data (175.4 cells/microcarrier).

### 3.7. Virus Sensitivity

**Figure 9:**
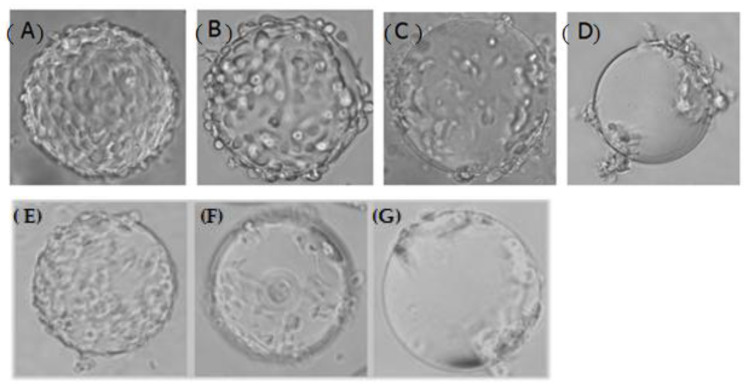
Cytopathic effect after infection with ISKNV (**A**–**D**) or SCRV (**E**–**G**) observed under the microscope (40×). (**A**) No obvious variation appeared at the first 2 days post infection (d p.i) with ISKNV; (**B**) Infected cells were enlarged and clustered at 3–4 d p.i.; (**C**) CPE appeared in more than 90% of the cells at 5 d p.i.; (**D**) Most of the cells detached from the microcarriers; (**E**) Cell shrinkage and rounding were observed at 12 h p.i.; (**F**) Some cells detached from the microcarriers at 24 h p.i., drawbench and plaque appeared; (**G**) Most of the cells detached from the microcarriers.

The suspended CPB cells were tested for sensitivity to ISKNV and SCRV infection. During the first 2 days post-infection (p.i.) with ISKNV, there was no obvious variation (Figure 9A). Then, cell shrinkage and rounding were observed at 3–4 d p.i. Gradually, infected cells were enlarged and clustered, and some of the cells began to detach from the microcarriers (Figure 9B). On the fifth day, CPE appeared in more than 90% of the CPB cells (Figure 9C) and almost all of the cells finally detached from the microcarriers (Figure 9D).

While for SCRV infection, cell shrinkage and rounding were observed at 12 h p.i. (Figure 9E). Then, some cells detached from the microcarriers and drawbench appeared between cells at 24 h p.i. (Figure 9F). At last, more than 90% of the cells detached within 48 h p.i. (Figure 9G).

## 4. Discussion

Microcarrier technology, providing a large surface area for cell growth, has become a popular platform for cell production [14,15,21,22,23,24]. However, few reports have referred to cells of aquatic animals [25,26,27,28]. As one of the most frequently-used commercial microcarriers, Cytodex 1 could provide attachment growth surface for lots of animal cells [14,15,20,29]. Data in our current study has also proved that CPB cells could grow well on this kind of microcarrier under a suitable stirring regime. To our knowledge, this is the first report on microcarrier-based bioreactor expansion of this type of cells.

The initial distribution of attached cells was recognized as the most critical stage, which was used to evaluate attachment efficiency [30,31,32]. Suitable intermittent agitation could enhance the contact time and also the attachment rate. Agitation ensured that cells and microcarriers are distributed homogeneously in the culture medium, while standing aims to facilitate steady cell attachment to the microcarrier. Both procedures were neither too short nor too long. A long-term constant agitation at the initial stage after inoculation will result in a low attachment rate or unstable attachment of cells to the microcarriers. Alfred et al. [19] reported that cell/microcarrier suspensions could be agitated intermittently for 3 min every half an hour for 5 h and then continued in static or stirred culture to facilitate cell attachment to microcarriers. In our study, 3 min/42 min/5 h were determined as the optimal agitation method, with which can obtain a high attachment rate to the microcarriers quickly and a homogeneous cell distribution.

After the initial attachment, the subsequent agitation rate is another important factor for CPB cells growth on Cytodex 1. To ensure the availability of sufficient nutrients and oxygen for the cells, microcarriers are often suspended by agitation to supply a flowing culture environment [14,33]. Brindley et al. [34] reported that mild flow shear stress could promote proliferation, whereas increasing levels of shear force above a certain threshold promotes differentiation at the expense of proliferation and self-renewal. In Ma‘s study [35], low levels of shear were found to affect proliferation as well as cytokine production of MSCs. Microcarriers may distribute heterogeneously and even settle at the bottom when agitated slowly. In this case, cells will become agglomerated in a bad status and could not proliferate regularly. Sun et al. [21] reported that the collision probability of shearing force between microcarriers increased when the agitation rate was enhanced and may result in a large number of damaged cells. In our study, taking the 125 mL flask as an example, cells could not reach a maximum when the agitation rate was increased to 50 r/min. Furthermore, it is well-known that the volume of the culture system also affects the agitation effect. Theoretically, the larger the culture system, the faster the optimal agitation rate that will be needed. The data from the present study demonstrate that the optimal agitation rates are 40, 45 and 55 r/min for 125 mL and 500 mL stirring flasks and 3 L bioreactors, respectively.

The density of living cell inoculation means the ratio of number of inoculated cells to microcarriers. The number of the inoculated cells plays an important role in the cell growth [36]. With low inoculation density, cells are distributed heterogeneously on the microcarriers and transmission of the growth signal is limited. In this case, high cell multiplication ratios may appear, but at the cost of a large number of unpopulated microcarriers and a low final cell density [30,37]. Our data proved that when inoculated at 20 cells/microcarrier, the empty-loading ratio was high, which is in line with previous reports, while with a high inoculation density such as 40–50 cells/microcarrier, the attachment surface and nutrient substance may be limited. Meanwhile, nutrient substances are consumed, metabolites accumulate quickly, and the pH declines rapidly. Hence, a dexterous culture environment is created.

Microcarrier concentration was found to affect cell growth. In order to save cultivation cost, the concentration is generally increased as high as possible. Some reports have shown that the concentration of microcarriers was proportional to the density of cells and the viral titer within a certain range [20,29]. However, cytotoxicity accompanied the deteriorated culture medium environment. This is due to the fact that the higher the concentration of the microcarrier, more cell metabolites will be produced, leading to the faster consumption of culture medium and a deleterious growth environment. Moreover, large numbers of damaged cells will appear because of the shear force produced by probability of the increased collisions between microcarriers. Our data validated that an increase of the microcarrier concentration could not enhance the cell expansion rate. Taking into consideration the cost of the culture medium, labor and time consumption, 3 g/L is the optimal microcarrier concentration. With these parameters cells are distributed homogeneously and could achieve a maximum density.

For the large-scale cultivation process, it is difficult to inoculate cells into a large bioreactor system directly from static culture cells. Instead, this should be performed step by step. Trypsin digestion is the popular approach for removing cells from microcarriers [14,38,39,40]. In our study, most of the cells detached from old microcarriers, and re-attached to new microcarriers after being resuspended. However, trypsin digestion could also damage cytomembranes and even cause the death of some cells, resulting in bad effects on viability and proliferation capacity. In order to investigate the feasibility of amplification for CPB cells cultured on Cytodex 1, several ratios from 1:2 to 1:5 were tested. With the four amplification ratios, all of the cell densities declined on the first day, which is similar to Yin’s results [40]. Perhaps there is some damage to the cytomembrane which weakens the attachment ability of cells. As a result, only 50–70% of the cells survied. Fortunately, most of the cells re-attached to the microcarriers and could repair well 24 h later proving the feasibility of expansion of CPB cells grown on Cytodex 1.

With the optimal parameters, CPB cells grew well on Cytodex 1, which indicated that the bioreactor expansion process yielded large quantities of CPB cells with similar characteristics to a conventional static culture system. Furthermore, the suspended cells were sensitive to ISKNV and SCRV. In conclusion, we have developed a high-yield process for proliferation of CPB cells and it will make preparations for large-scale production of ISKNV and SCRV vaccines. Parameters such as viral inoculation time, multiplicity of infection and harvest time will be studied in our subsequent research.

## Figures and Tables

**Table 1:** Agitation conditions at the cell attachment stage.

Agitation Conditions	A	B	C	D	E	F	G	H	I
Stirring time/min	3	3	3	3	3	3	3	3	3
Standing time/min	27	27	27	42	42	57	57	57	57
Total time/min	120	300	480	120	300	480	120	300	480

A~I represent different intermittent stirring method with low agitation rate at the early stage of cell attachment.

**Table 2:** Cell expansion fold and specific growth rate at different microcarrier concentrations.

Microcarrier Concentration (g/L)	2	3	4	5
^1^ Cell expanding fold	5.91 ± 0.03	5.85 ± 0.05	5.18 ± 0.01	4.45 ± 0.09
^2^ Specific growth rate	0.58 ± 0.03	0.57 ± 0.01	0.55 ± 0.03	0.50 ± 0.02

^1^ Cell expanding fold = the maximum cell density/the density of inoculated cells. ^2^ The mean of the daily cell specific growth rate μ_n_ in the exponential phase is the cell specific growth rate in the exponential phase. μ_n_ = (LnX_n_ − LnX_n−1_)/(t_n_ − t_n−1_), X represents the cell concentration, t represents incubation time, n and n − 1 represent the point of time for sampling and counting.

## Data Availability

Data available upon request.
